# CD4+ T cells facilitate replication of primary HIV-1 strains in macrophages and formation of macrophage internal virus-containing compartments

**DOI:** 10.1128/jvi.00182-25

**Published:** 2025-03-25

**Authors:** Sabina Victoria, Johanna Leyens, Lea Marie Meckes, Georgios Vavouras Syrigos, Gabriela Turk, Michael Schindler

**Affiliations:** 1 Institute for Medical Virology and Epidemiology of Viral Diseases, University Hospital Tübingen 493697 , Tübingen, Germany; 2 CONICET–Universidad de Buenos Aires, Instituto de Investigaciones Biomédicas en Retrovirus y SIDA (INBIRS) 28196 , Buenos Aires, Argentina; 3 Departamento de Microbiología, Parasitología e Inmunología, Facultad de Medicina, Universidad de Buenos Aires 62882 , Buenos Aires, Argentina; Icahn School of Medicine at Mount Sinai, New York, New York, USA

**Keywords:** HIV-1, macrophages, transmitted-founder HIV-1, virus-containing compartments, VCC, primary HIV-1 strains, cell-to-cell transmission, viral persistence, viral latency

## Abstract

HIV-1 replication in macrophages is highly variable with internal virus accumulation in so-called virus-containing compartments (VCCs). VCCs represent a reservoir that is shielded from the antiviral immune response. VCC formation has been studied in lab-adapted HIV-1, but it has not been investigated whether primary HIV-1 strains induce VCCs. Furthermore, although macrophages transmit HIV-1 from VCCs to CD4+ T cells, the effect of T cells on VCCs is unknown. We analyzed the ability of primary and lab-adapted HIV-1 to replicate in macrophages, the effect of non-infected CD4+ T cell coculture, and VCC formation. All HIV-1 strains replicated in CD4+ T cells, whereas only lab-adapted HIV-1 replicated efficiently in macrophage monocultures. Coculture with non-infected CD4+ T cells enhanced the replication of primary HIV-1 in macrophages, a process associated with increased VCC formation and dependent on direct cell-to-cell contact. Broadly neutralizing antibodies differentially affected CD4+ T cell-mediated enhancement of HIV-1 replication in macrophages. CD4 antibody treatment of macrophages phenocopied the infection-promoting effect of CD4+ T cell coculture. In conclusion, non-infected CD4+ T cells facilitate primary HIV-1 replication in macrophages, and the induction of VCCs appears to be a proxy for this phenotype. VCC formation and HIV-1 replication in macrophages are promoted by non-infected CD4+ T cells in a CD4- and GP120-dependent manner. Our findings highlight the critical role of T cell-macrophage interaction in HIV-1 replication dynamics and VCC formation and call for strategies to interfere with VCCs in order to target the HIV-1 reservoir in macrophages.

**IMPORTANCE:**

Here, we focus on the intimate interplay between HIV-1-infected macrophages and CD4+ T cells. Specifically, we analyzed whether primary HIV-1 strains induce virus-containing compartments (VCCs) within macrophages, which are thought to serve as viral sanctuaries and macrophage reservoirs. Notably, primary HIV-1 strains were unable to replicate in macrophages and induce VCCs unless they were cocultured with non-infected CD4+ T cells, leading to enhanced VCC formation and viral replication. This suggests an essential role for non-infected CD4+ T cells in facilitating primary HIV-1 replication in macrophages. Our data highlight the importance of not only addressing the latent HIV-1 T cell reservoir but also targeting VCC formation in macrophages to achieve the ultimate goal of functional HIV-1 cure.

## INTRODUCTION

Human immunodeficiency virus (HIV) has spread worldwide since its first identification as the causative agent of acquired immune deficiency syndrome (AIDS) in 1983 (1). More than 85 million people have been infected with HIV, and 40 million people have died worldwide due to AIDS-related illnesses (2). HIV primarily infects CD4+ T cells, monocytes, macrophages, and dendritic cells since these cells express the CD4 receptor, as well as the co-receptors CCR5 or CXCR4, which are required for its entry into target cells (3). Upon prolonged HIV-1 infection, there is a drastic depletion of infected and activated CD4+ T cells, which leads to the loss of cell-mediated immunity, rendering people living with HIV-1 (PLWH) susceptible to fatal opportunistic infections that are defined as AIDS.

Global scientific efforts to combat AIDS have led to the development of efficient antiretroviral therapy (ART) regimens, which suppress viral replication by targeting various steps of the viral life cycle. By decreasing the plasma viral load in patients and preventing disease progression, antiretroviral therapy restores the life expectancy of the patients to an almost normal level (4, 5). Although ART is highly beneficial in controlling HIV infection, it requires lifelong administration and has various side effects. To date, an HIV vaccine has not been developed, and there is no cure available that can eradicate the virus from an infected individual. Therefore, there is an ongoing need to understand HIV-1 persistence and transmission in order to devise novel prophylactic and therapeutic treatment strategies (6).

Macrophages are known to be among the first cells that encounter HIV-1 during sexual transmission. They phagocytose pathogens or particles in submucosal tissues and transport them to draining lymph nodes, where they present the antigens to CD4+ T cells and activate CD8+ cytotoxic T cells, initiating immune control of the infection (7, 8).

Upon the interaction of a CD4+ T cell with an antigen-presenting cell (APC), adhesion molecules on the surface of CD4+ T cells probe the APC surface. Recognition of antigen peptide/MHC-II complex leads to the formation of an immunological synapse by concentration of T cell receptors, CD4, and other signaling molecules. This event stabilizes the cell contact, allowing T cell activation and proliferation signals to be sustained (9).

Macrophages can be productively infected by HIV-1 and serve as a reservoir, even in PLWH under ART (8, 10, 11). Infected macrophages sequester the virus within an intracellular plasma membrane compartment that is protected from the extracellular milieu (12, 13). These HIV-1-containing compartments in macrophages (VCCs, virus-containing compartments) were initially thought to be late endosomes, respectively, multivesicular bodies. However, high-dimensional microscopy, including electron microscopy, showed that these compartments are characterized by a complex three-dimensional structure, contain unique protein markers, including tetraspanins CD81, CD9, and CD53, have a near-neutral pH, and are composed of invaginated plasma membrane folds that often remain connected to the cell surface (12, 14–17). HIV-1 can be rapidly translocated from macrophage internal VCCs via the so-called virological synapse to infect T cells (18–20).

Most studies that investigate HIV transmission and pathogenesis take advantage of the availability and convenience of laboratory-adapted HIV-1 strains. Although they are easy to employ as they efficiently replicate in cell lines, lab-adapted HIV-1 might have lost important features of primary HIV-1 strains. On the other hand, primary HIV-1 strains more closely resemble HIV-1 infection in PLWH since they are directly isolated from plasma specimens. Notably, in more than 80% of the sexual transmission events of HIV-1, a single virus is transmitted from the donor to the recipient, despite the diversity present in the donor (21). This virus initiates the infection in the recipient, and the virus isolated from PLWH during the first 6 months of infection is therefore called a transmitted/founder (T/F) virus. As the infection progresses to the chronic stages, the virus population in the blood diversifies due to mutations and selection pressure caused by the adaptive immune responses of the host. The virus isolated from the PLWH’s blood at this period is called a chronic virus (22).

Studies have evidenced HIV-1 transfer from macrophages to uninfected T cells and addressed the important role of VCCs in this process (18–20, 23–27). However, most of these studies rely on lab-adapted HIV strains. Some studies using T/F strains have examined infection dynamics but have not thoroughly explored the mechanisms of cell-to-cell transmission (28, 29). Others have shown that monocyte-derived macrophage (MDM) infection with T/F strains occurs more efficiently upon contact with infected CD4 T cells, but it is still unclear whether the reverse is also true (30–32). Thus, more investigations are required to further understand the modes of primary HIV-1 strain replication and transmission in *in vivo* relevant target cells, especially the interplay and virus transmission between infected MDMs and uninfected CD4 T cells. To address this, we employed a set of T/F and chronic HIV-1 strains to evaluate their infection efficiency in primary macrophages and the impact of autologous CD4+ T cell coculture. We also assessed the efficiency of VCC formation by these isolates, as investigations into VCC formation and their involvement in macrophage-to-T cell transmission have predominantly relied on laboratory-adapted viral strains, such as HIV-1 NL4-3 and AD8.

## MATERIALS AND METHODS

### Cell lines

HEK-293T cells (DSMZ ACC635) were maintained in Dulbecco’s modified Eagle’s medium (high glucose, GlutaMAX supplement, Gibco) supplemented with 10% heat-inactivated fetal calf serum (FCS; Invitrogen), 100 µg/mL streptomycin, and 100 units/mL penicillin (Sigma) and were cultured at 37°C, 90% humidity, and 5% CO_2_.

### HIV-1 constructs

Infectious molecular clones pHIV-1 AD8, pYK-JRCSF, and pBR-NL43-V3 92th014.12 have been described (15), as well as pHIV-1 CH077, pHIV-1 THRO, pHIV-1 CHO58 (33), and pUC57rev_HIV-1 C CH293 (22).

### Generation of virus stocks

To produce HIV-1 stocks, 0.5 × 10^6^ HEK-293T cells were seeded per well in a 6-well plate, and 1 day later, the cells were transfected using the calcium phosphate method with 5 µg of the respective HIV-1 molecular clone or mock transfected. At 16 h post-transfection, the medium was changed, and supernatants containing the HIV-1 stock were collected 24 h later and cleared by centrifugation at 3,200 *g* at 4°C for 10 minutes. HIV-1 stocks were quantified by an in-house p24 enzyme-linked immunosorbent assay (ELISA) immediately after virus stock collection.

To generate VPX-VLPs (SIV VPX-containing virus-like particles), the above-mentioned protocol was followed, and HEK-293T cells were transfected with 5 µg of pSIV_Vpx1 (34) and cotransfected with 0.5 µg of pHIT-G (vesicular stomatitis virus G protein). At 16 h post-transfection, the medium was changed, and supernatants containing the VLP stock were collected and cleared 24 h later.

### Isolation and differentiation of primary human monocyte-derived macrophages

Monocyte-derived macrophages (MDMs) were generated from buffy coats following established protocols (15, 35). Briefly, peripheral blood mononuclear cells (PBMCs) were isolated from buffy coats by Ficoll-Paque PLUS (GE) density gradient centrifugation. Subsequently, 2.0 × 10^7^ PBMCs were seeded in petri dishes (Greiner Bio-One) in RPMI 1640 (GlutaMAX Supplement; Gibco) medium supplemented with 4% human AB serum (Sigma), 2 mM L-glutamine (PAA), 100 µg/mL streptomycin, and 100 units/mL penicillin (Sigma), 1 mM sodium pyruvate (Gibco), 1× non-essential amino acids (Invitrogen), and 0.4× minimal essential medium vitamins (Biochrom). Monocytes were differentiated for 3 days by plastic adherence into MDM. Following this initial differentiation phase, non-adherent cells were removed by washing, and the MDMs were further differentiated for 4 days. Accutase (Sigma) was used to detach the MDM for 45 minutes at 37°C.

### Isolation and maintenance of primary human CD4+ T cells

To isolate CD4+ T cells from buffy coats, the RosetteSep Human CD4+ T Cell Enrichment Cocktail (StemCell) was used according to the manufacturer’s instructions. CD4+ T cells were cultured in RPMI-1640 (GlutaMAX Supplement; Gibco) medium supplemented with 10% FCS (Invitrogen), 100 µg/mL streptomycin, and 100 U/mL penicillin (Sigma), containing 10 ng/mL interleukin-2 (IL-2, = 4.1×10² IU/mL; StemCell). Cells were maintained under standard conditions at 37°C, 90% humidity, and 5% CO_2_. Prior to infection, T cells were expanded and activated for 3 days using RPMI (GlutaMAX Supplement; Gibco) medium supplemented with 10% FCS (Invitrogen), 100 µg/mL streptomycin, and 100 U/mL penicillin (Sigma), containing 10 ng/mL IL-2 (=4.1 × 10² IU/mL; StemCell) and 1 µg/mL lectin from *Phaseolus vulgaris* (red kidney bean, PHA; Sigma).

### MDM and CD4+ T cell infection

For MDM and CD4+ T cell infection, MDMs were seeded (5,000 per well) in a 96-well plate (Greiner, flat bottom black polystyrene wells with micro-clear bottom). Twenty-four hours later, cells were transduced with VPX-containing VLPs for 2 h before infection. Pre-stimulated primary CD4+ T cells were seeded (200,000 cells per well) in a 96-well plate, U bottom (Greiner). Cells were infected for 16 h (MDM) and 4–6 h (CD4 +T cells) with 125, 250, and 500 ng/mL of p24 of the HIV-1 strains mentioned in “HIV-1 constructs and generation of HIV-1 stocks.” At 4 dpi, the MDM media were replenished, and at 8 dpi (for MDM) and 4 dpi (for CD4 +T cells), HIV-1 replication was analyzed in the cells as well as in the supernatant (SUP).

### MDM and CD4+ T cell infection in coculture

MDMs were seeded (5,000 per well) in a 96-well plate (Greiner, flat bottom black polystyrene wells with micro-clear bottom). Twenty-four hours later, cells were transduced with VPX-containing VLPs for 2 h before infection. Subsequently, the cells were infected for 16 h with 125, 250, and 500 ng/mL of p24 of the respective HIV-1 strains. At 4 dpi, PHA and IL-2 pre-stimulated CD4+ T cells were added to the macrophage culture at a 1:10 ratio (1 MDM: 10 CD4+ T cells). HIV-1 replication was analyzed in CD4+ T cells, MDM, and the SUP at 8 dpi.

### MDM and CD4+ T cell infection in coculture under ART, bnAB, and a-CD4 mAB conditions

MDMs were seeded (8,000 per well) in a 96-well plate (Greiner, flat-bottom black polystyrene wells with micro-clear bottoms). After 24 h, cells were transduced with VPX-containing VLPs for 2 h before infection. MDMs were then infected for 16 h with 250 ng/mL of p24 from the respective HIV-1 strains. At 4 dpi, MDMs were preincubated with either 10 µM Raltegravir (Sigma) or 10 µM Nevirapine (Merck), or DMSO (Sigma) as a control; 10 µg/mL 3BNC117, PG9, and 10-1074 (NIH HIV Reagent Program); 10 µg/mL InVivoMAb human IgG1 isotype control (Biozol); or 10 ng/mL anti-CD4 mAb (BioXCell, clone: RPA-T4, mouse derived); or 10 µg/mL mouse IgG1, κ isotype control (BioLegend), respectively. After 2 h of preincubation, CD4+ T cells (pre-stimulated with 10 ng/mL IL-2 [=4.1 × 10² IU/mL] and 1 µg/mL PHA for 3 days or maintained with 10 ng/mL IL-2 alone) were added to the macrophage culture at a 1:10 ratio (1 MDM: 10 CD4+ T cells). The concentration of these treatments remained constant throughout the experiment, and the coculture was maintained under the respective conditions until the harvest time point. HIV-1 replication was analyzed in CD4+T cells, MDM, and the SUP at 8 dpi.

### MDM and CD4+ T cell infection in coculture under transwell condition

MDM were seeded (50,000 per well) in a 24-well plate (Corning, Transwell 24 well plates). After 24 h, cells were transduced with VPX-containing VLPs for 2 h before infection. MDMs were then infected for 16 h with 250 ng/mL of p24 from the respective HIV-1 strains. At 4 dpi, PHA and IL-2 pre-stimulated CD4+ T cells were added either directly to the MDM or placed in a transwell insert (0.4 µm pore size) within the macrophage culture at a 1:10 ratio (1 MDM: 10 CD4+ T cells). HIV-1 replication was analyzed in CD4+ T cells, MDM, and SUP at 8 dpi.

### Quantification of HIV-1 replication in MDM

Upon removal of the CD4+ T cells in the cell culture supernatants, the adherent MDMs in the cultures were extensively washed with PBS to remove any remaining T cells. After that, the MDMs were fixed with 2% PFA for 10 minutes at 37°C, permeabilization was performed with 1% Saponin-PBS for 10 minutes at RT, and blocked with PBS + 10% FCS for 20 minutes. Then, cells were stained with primary anti-HIV-Gag antibody (Beckman-Coulter; KC57-RD1, mouse derived) and anti-mouse Alexa Fluor555-conjugated secondary antibody (Invitrogen; goat derived). DAPI was used for nuclear staining and control for remaining T cells in the microscopic analysis. Finally, cells were washed and fixed with 2% PFA. Fluorescence microscopy images (4× and 40× magnification) were acquired with the Cytation 3 Cell Imaging Multi-Mode Reader (BioTek Instruments). Macrophage-residing HIV-1 in VCCs was analyzed with 40× magnification images. Filters used were the following: “Blue filter:” LED 405 nm, emissions maximum/bandwidth: 460/60 nm. “Green filter:” LED 505 nm, emissions maximum/bandwidth: 542/27 nm; and “Red filter:” LED: 590 nm, emissions maximum/bandwidth: 647/57 nm. Images were analyzed using Gen5 Software (version 5.3.10).

### High-resolution imaging of MDM

MDMs were seeded (150,000 per well) in a 12-well plate with added cover slips (High Precision Cover Slip, 18 mm diameter, VWR). After 24 h, cells were transduced with VPX-containing VLPs for 2 h before infection. MDMs were then infected for 16 h with 250 ng/mL of p24 from the respective HIV-1 strains. At 4 dpi, either the media of the MDM was replenished for monoculture or PHA and IL-2 pre-stimulated CD4+T cells were added to the macrophage culture at a 1:10 ratio (1 MDM: 10 CD4+ T cells) for coculture conditions. At 8 dpi, cells were washed extensively with PBS and fixed with 2% PFA for 30 minutes at 4°C. Permeabilization was performed with 1% Saponin-PBS for 10 minutes at RT, followed by blocking with PBS + 10% FCS for 20 minutes. Cells were then stained with anti-HIV-Gag antibody (Beckman-Coulter; KC57-FITC, mouse derived) and primary anti-CD81 antibody (Abcam; EPR21916, rabbit derived), followed by staining with secondary anti-rabbit Alexa Fluor 633-conjugated secondary antibody (Invitrogen; goat derived). DAPI was used for nuclear staining and control for remaining T cells in the microscopic analysis. Between and after staining steps, cells were washed three times with PBS for 5 minutes each. Stained macrophages were mounted on microscopy slides using VectaShield (Biozol), sealed with nail polish, and imaged using the DeltaVision OMX SR microscope (General Electric). Images were analyzed using IMARIS 9.3.0 (Bitplane, part of Oxford Instruments).

### Quantification of HIV-1 replication in CD4+ T cells

After the CD4+ T cells were fixed with 2% PFA for 10 minutes at 37°C, permeabilization was performed with ice-cold 90% methanol in H_2_O for 20 minutes on ice, followed by a blocking step with PBS + 10% FCS for 20 minutes. Then, cells were stained with primary anti-HIV-Gag antibody (Beckman-Coulter; KC57-FITC, mouse derived), and cells were acquired using the MACSQuant VYB Analyzer (Miltenyi) equipped with a 488 nm laser and a band filter (525/50). Data analysis was performed using Flow Logic (Flow Logic) software.

### Quantification of HIV-1 release in supernatant

HIV-1 p24 capsid antigen levels were quantified using an in-house-developed p24 ELISA. Virus stocks or cell culture supernatants were lysed with 1% Triton X-100 for 1 h at 37°C. Then, 96-well plates (ThermoFisher, NUNC Immuno Plate Maxisorp) were coated with anti-HIV-1 p24 monoclonal antibody (ExBio, clone MAK183, mouse derived) overnight, followed by washing and blocking with 10% FCS in PBS for 2 h at 37°C. A standard curve was generated using HIV-1 p24 capsid protein (Abcam; ab43037) in a 1:2 dilution series (50–97 pg/mL). Samples were diluted in PBS-T containing 0.05% Triton X-100 and incubated for 2 h at 37°C. After washing, primary anti-HIV-1 p24 antibody (Eurogentec, rabbit derived) was added, followed by secondary anti-rabbit-peroxidase antibody (Dianova). SureBlue TBM Peroxidase Substrate (SeraCare) was used for detection, and the reaction was stopped by adding 0.5 M H_2_SO_4_ (Applichem). The absorbance was measured at 450 and 650 nm using a Cytation Multiplate Reader (Biotek). Absolute p24 concentrations (ng/mL) were calculated based on the standard curve.

### Quantification of SamHD1 protein expression in MDM

MDMs were seeded (150,000 per well) in a 12-well plate (Not Treated Multiwell Plate, Falcon) or (50,000 per well) a 24-well plate (Not Treated Multiwell Plate, Falcon). After 24 h, cells were transduced with VPX-containing VLPs for 2 h. At 4 dpi, either CD4+ T cells (pre-stimulated with 10 ng/mL IL-2 and 1 µg/mL PHA for 3 days or maintained with 10 ng/mL IL-2 alone) were added to the macrophage culture at a 1:10 ratio (1 MDM: 10 CD4+ T cells) or MDMs were treated with 10 µg/mL anti-CD4 mAb (BioXCell, clone: RPA-T4, mouse derived) or 10 µg/mL mouse IgG1 and κ isotype control (BioLegend), respectively. After 4 days, MDM were fixed with 2% PFA for 10 minutes at 37°C, permeabilization was performed with ice-cold 90% methanol in H_2_O for 20 minutes on ice, followed by a blocking step with PBS + 10% FCS for 20 minutes. Then, the cells were stained with rabbit anti-SAMHD1 Ab (ProteinTech, dilution 1:1,000) or rabbit anti-pSAMHD1 Ab (specific for T592 phosphorylation, ProSci; dilution 1:1,000) antibody for 1 h at RT. After washing once, the cells were stained with goat anti-rabbit Alexa Fluor 555 (Thermo, 1:1,000) essentially as described before (36). MDMs were acquired using the MACSQuant VYB Analyzer (Miltenyi) equipped with a 488 nm laser and a band filter (525/50). Data analysis was performed using Flow Logic (Flow Logic) software.

### Statistical analyses

All statistical calculations were performed using GraphPad Prism (version 9.4.1). Unless otherwise stated, data are shown as the mean of at least three independent experiments ± SEM. Simple linear regression analysis was performed for the correlation studies, and the Pearson correlation coefficients, as well as *P*-values are shown.

## RESULTS

### CD4+ T cells but not macrophages support the replication of primary patient-derived HIV-1 strains

**Fig 1:**
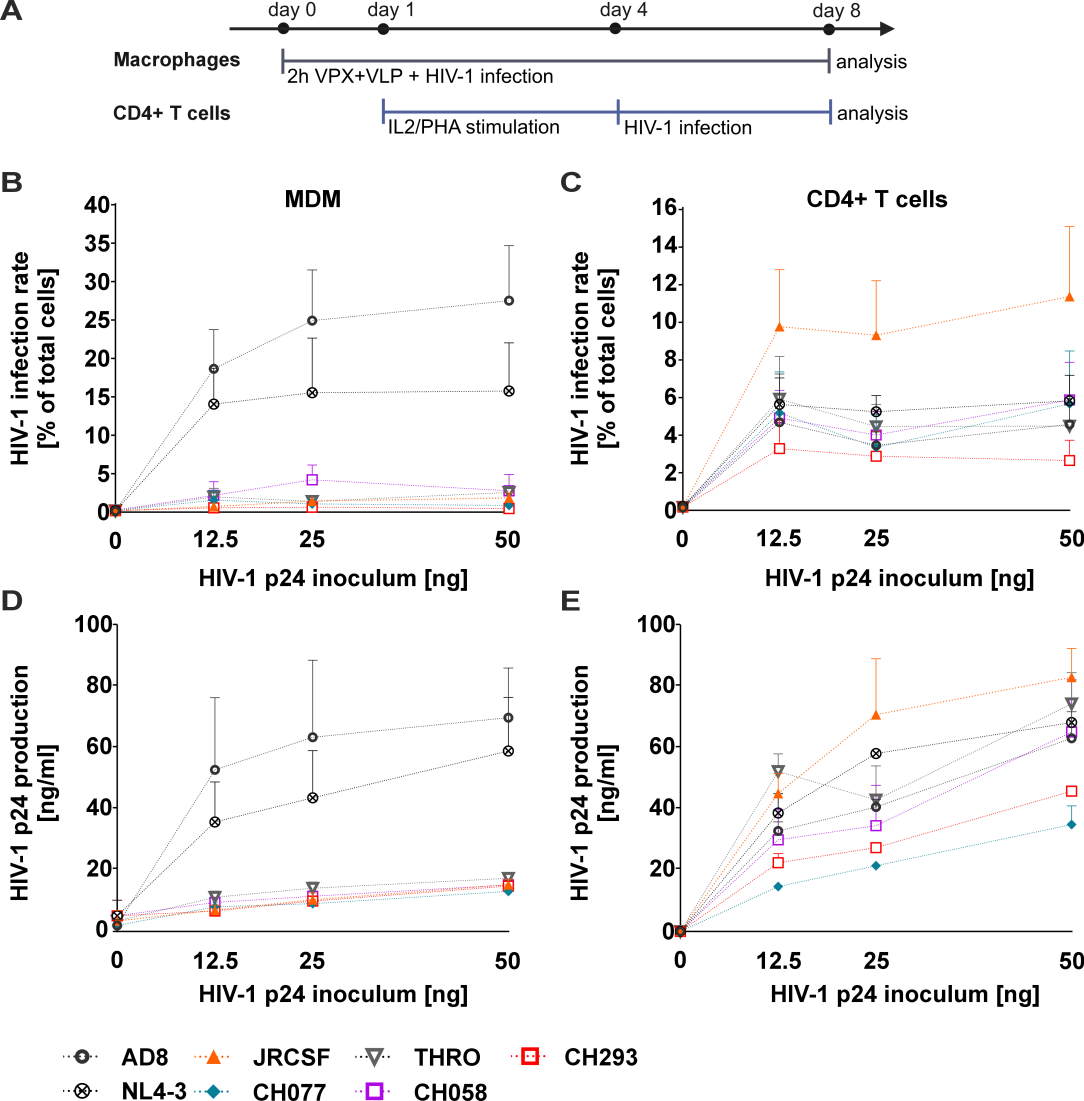
Primary HIV-1 strains replicate in CD4+ T cells but not in MDM. (A) Schematic representation of experimental design. On day 0, MDMs were pretreated for 2 h with VPX-containing VLPs, followed by infection with different HIV-1 strains for 8 days. Supernatants and MDMs were collected to assess HIV-1 production and infection rate, respectively. Stimulated primary CD4+ T cells were HIV-1 infected on day 4, and 4 days later, SUP and CD4+ T cells were harvested to assess HIV-1 production and infection rate, respectively. (B) MDM or (C) activated CD4+ T cells were infected with 12.5, 25, or 50 ng of HIV-1 p24 protein (inoculum) for 16 or 4 h, respectively. MDMs were immunostained to detect HIV-1 p24 and DAPI and analyzed by fluorescence microscopy at 8 dpi. The CD4+ T cells were immunostained to detect HIV-1 p24 and analyzed by flow cytometry at 4 dpi. HIV-1 replication is shown as infection rate and expressed as the percentage of (B) p24+/total MDMs or (C) CD4+ T cells, respectively. The concentration of HIV-1 p24 in cell culture SUP was evaluated by p24 ELISA at (D) 8 dpi for MDM or at (E) 4 dpi for CD4+ T cells, respectively. Data from *n* = 4 donors for AD8, NL4-3, JRCSF, CH077, THRO, and CH293 and *n* = 3 donors for CH058 are plotted as mean ± SEM.

We first aimed to analyze the basic characteristics of virus infection and replication of varying HIV-1 strains in CD4+ T cells and macrophages. For this, lab-adapted strain AD8, which is macrophage tropic (37, 38), and a CCR5-tropic version of NL4-3 (15, 39) were employed. Furthermore, we utilized the CCR5-tropic JRCSF, which was isolated from the cerebrospinal fluid of a chronic HIV-1 patient and classified as low- or non-macrophage tropic (37, 40), as well as four different primary HIV-1 strains that are all capable of using CCR5 as an entry receptor (22, 33). For a robust readout, macrophages were pre-treated for 2 h with SIV VPX-VLPs (41, 42). This inactivates the known HIV-1 restriction imposed by SAMHD1 (43), allowing the assessment of SAMHD1-independent mechanisms that may affect the infection of macrophages by primary patient-derived HIV-1. Then, macrophages were infected with the different HIV-1 strains using increasing amounts of virus normalized to the incoming amount of p24, and the efficiency of virus replication and spread in the culture was assessed 8 days later by p24-immunofluorescence staining to identify infected cells (Fig. 1A). While the lab-adapted R5-tropic AD8 (~25% p24+ cells) and NL4-3 (~15% infected cells) showed a high proportion of infected cells at that time point, as expected, the primary HIV-1 strains were severely impaired in their capability to cause efficient infection and spread (less than 1% of infected cells, compare Fig. 1B). Only the primary isolate CH058 showed evidence of low-level infection and replication, with 2%–4% of infected macrophages.

In contrast, replication of all strains was readily detectable in CD4+ T cells analyzed at 4 days post-infection (Fig. 1C). We chose this earlier time point compared to macrophages since there is spread and replication in the culture occurring prior to the onset of massive virus-induced cytotoxicity and activation-induced cell death (44, 45). In order to corroborate the measurement done on infected cells, we assessed virus replication by quantifying p24 production in cell culture supernatants over 8 days (macrophages, Fig. 1D) or 4 days (CD4+ T cells, Fig. 1E), which is an indicator of the overall efficiency of virus production in the cultures. Consistent with our previous observations, only lab-adapted AD8 and R5-tropic NL4-3 were efficiently released from infected macrophages, whereas all other strains showed low signs of p24 production (Fig. 1D). In contrast, CD4+ T cells sustained p24 release across all tested strains (Fig. 1E). In conclusion, while it was expected that the primary strains replicate in CD4+ T cells, albeit with differential efficacy, primary strains capable of utilizing CCR5 for entry are strongly compromised in terms of macrophage infection and p24 production in this model.

### Coculture of HIV-1-infected macrophages with CD4+ T cells facilitates viral replication

**Fig 2:**
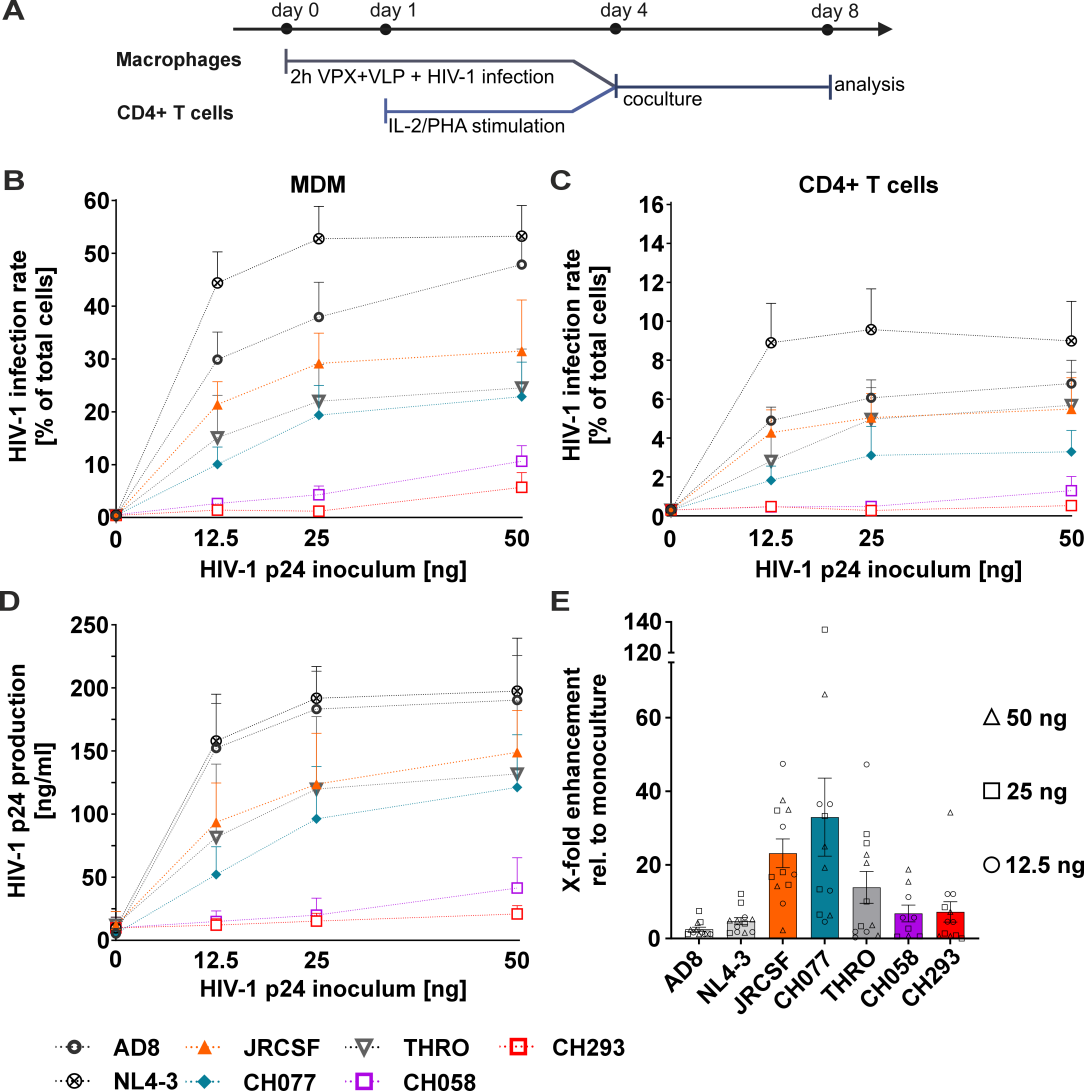
CD4+ T cells facilitate replication of chronic and T/F isolates in MDM. (A) Schematic representation of experimental design. On day 0, MDMs were pretreated for 2 h with VPX-containing VLPs, followed by HIV-1 infection. Stimulated primary CD4+ T cells were cocultured with infected MDMs on day 4. At 4 days post-coculture, SUP, MDM, and CD4+ T cells were harvested to evaluate HIV-1 production and infection rates, respectively. (B) MDMs were infected with 12.5, 25, or 50 ng of HIV-1 p24 (inoculum) for 16 h, and washed and activated CD4+ T cells were added at 4 dpi at a ratio of 1:10 (1 MDM: 10 CD4+ T cells). MDMs were immunostained to detect HIV-1 p24 and counterstained with DAPI for fluorescence microscopy at 8 dpi. (C) The CD4+ T cells from the coculture were immunostained to detect HIV-1 p24 and analyzed by flow cytometry at 4 dpi. HIV-1 replication is shown as infection rate, expressed as the percentage of (B) p24+/ total MDMs or (C) CD4+ T cells, respectively. (D) The concentration of HIV-1 p24 in the coculture was measured via p24 ELISA at 8 dpi. (E) The X-fold enhancement of replication in MDMs is shown. Circles represent MDMs initially infected with 12.5 ng of HIV-1 p24, squares represent 25 ng, and rectangles represent 50 ng of HIV-1 p24 inoculum. Data from *n* = 4 donors for AD8, NL4-3, JRCSF, CH077, THRO, and CH293 and *n* = 3 donors for CH058 are plotted as mean ± SEM.

Macrophages and CD4+ T cells interact via various mechanisms, including direct cell-to-cell contacts, such as the immunological synapse, or indirectly by secretion of cytokines and chemokines. Overall, such non-virus-specific interactions seem to promote virus replication and spread and may be central to viral pathogenesis (46, 47). Furthermore, there is evidence for cytokine and chemokine secretion that might be both beneficial and inhibitory in the context of virus replication (48–51). However, most studies in this area have focused on infected CD4+ T cells cocultured with non-infected macrophages (30, 31, 52). Furthermore, especially for primary patient-derived HIV-1, there are few studies analyzing the overall effects of coculturing HIV-1-infected macrophages with non-infected CD4+ T cells (24, 25, 53, 54). To analyze this, we infected macrophages with the different HIV-1 laboratory-adapted and primary strains using increasing amounts of virus normalized to the amount of p24. After 16 h, macrophages were washed to remove virus inoculum. Three days later, activated CD4+ T cells were added and left in coculture for an additional 4 days (Fig. 2A). Then, after the removal of the CD4+T cells in the supernatants and extensive washing, we assessed the rates of macrophage infection and replication by p24 staining and counting of infected cells via automated medium-throughput fluorescence microscopy. CD4+ T cell infection was quantified via p24 staining and flow cytometry. The overall levels of virus replication and HIV-1 production in the cocultures were assessed in the supernatants by p24 ELISA.

**Fig 3:**
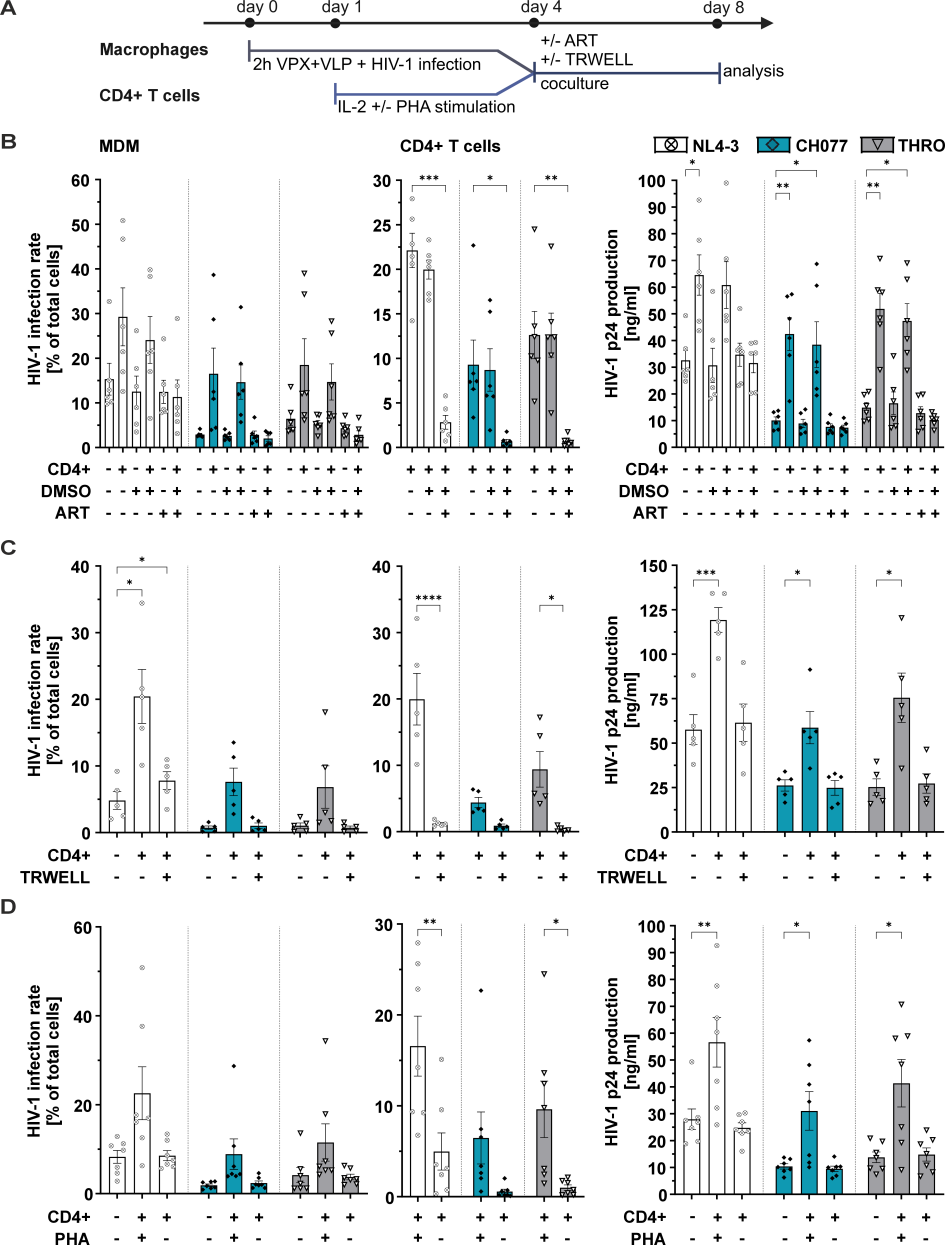
Cell-to-cell contact and T cell activation are required to enhance HIV-1 macrophage infection. (A) Schematic representation of the experimental design. Setup is similar to Fig. 2A; however, CD4+ T cells were either activated on day 1 with IL-2 and PHA or maintained with IL-2 alone. On day 4, CD4+ T cells were cocultured with infected MDMs at a 1:10 ratio (1 MDM: 10 CD4+ T cells) either directly or separated by transwell inserts. Additionally, in some conditions, MDMs were treated with antiretroviral therapy during the coculture. (B) The left panel shows the HIV-1 infection rate in MDMs cocultured with CD4+ T cells under ART treatment (10 µM nevirapine and 10 µM raltegravir). DMSO was used as a negative control. The middle panel presents the corresponding infection rates in the cocultured CD4+ T cells, while the right panel displays HIV-1 p24 production in the coculture SUP. (C) The left panel depicts the HIV-1 infection rate in MDMs cocultured with CD4+ T cells under conditions of direct cell-to-cell contact or separated by transwell (TRWELL) inserts (0.4 µm pore size). The middle panel shows the respective infection rates in the cocultured CD4+ T cells, while the right panel highlights HIV-1 p24 production in the coculture supernatants. (D) The left panel illustrates the HIV-1 infection rate in MDMs cocultured with either PHA-prestimulated CD4+ T cells or non-stimulated CD4+ T cells. The middle panel displays the infection rates in the cocultured CD4+ T cells, and the right panel shows HIV-1 p24 production in the coculture supernatants. Data from *n* = 6 donors for ART treatment, *n* = 5 donors for the TRWELL condition, and *n* = 7 donors for non-activated T cell conditions are presented as mean ± SEM. Statistical significance was assessed using a paired two-way ANOVA. Dunnett’s multiple comparisons test was applied for MDM and p24 values, while Sidak’s correction was used for CD4+ T cell data. (*P ≤ 0.05, **P ≤ 0.01, ***P ≤ 0.001, and ****P ≤ 0.0001).

Strikingly, coculturing the HIV-1-challenged macrophages with autologous non-infected CD4+ T cells strongly enhanced the levels of virally infected macrophages in the cultures (Fig. 2B). This was mirrored by CD4+ T cell infection rates (Fig. 2C) and p24 production in cell culture supernatants (Fig. 2D). R5-tropic lab-adapted strains AD8 and NL4-3 doubled the productive infection rate of macrophages (~25% without CD4+ T cells to ~45% with CD4+ T cells for AD8 and for NL4-3 from ~15% to ~50% with CD4 +T cells; compare Fig. 1B to Fig. 2B). Throughout, the effect was even more dramatic for the primary strains, as evident by the fold increase in infection rates when HIV-1-infected macrophages were cocultured with autologous non-infected CD4+ T cells (Fig. 2E). For instance, the HIV-1 T/F strain CH077 infected ~20% of macrophages at 50 ng of p24 inoculum when CD4+ T cells were added (Fig. 2B) compared to nearly no infection without the addition of CD4+ T cells (Fig. 1B). We made similar observations for the T/F strain THRO and CH058 and the chronic HIV-1 strain CH293 (compare Fig. 2B to Fig. 1B). Similarly, JRCSF achieved high infection rates of about 30% at 50 ng p24 in the inoculum (Fig. 2B), whereas the isolate was unable to replicate in macrophages without CD4+ T cell coculture (Fig. 1B). To corroborate that the enhancing effect exerted by coculturing HIV-1-infected macrophages with autologous non-infected CD4+ T cells is indeed increased viral replication, we repeated the experiment with the R5-tropic HIV-1 NL4-3 and the primary isolates CH077 and THRO in the presence of antiretroviral drugs (ART) (Fig. 3A and B). As expected, the addition of ART prevented the CD4+ T cell-induced increase in HIV-1-infected macrophages, HIV-1 transmission to CD4+ T cells, and increased p24 production in cell culture supernatants.

Altogether, primary patient-derived HIV-1 strains, which are severely impaired in their ability to productively infect macrophages, efficiently replicate and spread in these cells upon coculture with autologous non-infected CD4+ T cells.

### CD4+ T cells enhance HIV-1 replication in macrophages via cell-to-cell contact in a CD4- and GP120-dependent manner

We next analyzed the mechanistic determinants of how autologous non-infected CD4+ T cells enhance HIV-1 replication in macrophages (Fig. 3A). For this, we employed three HIV-1 strains, the lab-adapted R5-tropic NL4-3 and two primary isolates, CH077 and THRO. CD4+ T cells could possibly secrete soluble factors that enhance HIV-1 macrophage replication or boost productive infection via direct cell-to-cell contact. To clarify this, non-infected PHA-stimulated CD4+ T cells were cocultured with macrophages 4 days post-infection and left either in direct contact or separated via a transwell (Fig. 3C). Notably, when transwell inserts prevented direct contact of the HIV-1-infected macrophages with the non-infected CD4+ T cells, we did not observe any enhancement of macrophage infection nor did we see CD4+ T cell infection or increased p24 production in cell culture supernatants (Fig. 3C). In conclusion, CD4+ T cells need to directly interact with macrophages to increase HIV-1 infection, and macrophage-derived HIV-1 is primarily transmitted to CD4+ T cells via cell-to-cell contact.

Canonically, CD4+ T cells engage in macrophage interaction upon antigenic stimulation of the T cell-receptor (TCR) complex. We, therefore, analyzed if TCR stimulation is required for non-infected CD4+ T cells to increase HIV-1 replication in macrophages (Fig. 3D). CD4+ T cells were treated with IL-2 only or additionally TCR-stimulated with PHA for 3 days. Subsequently, the CD4+ T cells were cocultured with macrophages that were infected for 4 days with HIV-1 NL4-3 or the primary isolates CH077 or THRO. The number of infected macrophages and T cells was quantified 4 days later, along with p24 production in cell culture supernatants. Clearly, pre-stimulation of autologous non-infected CD4+ T cells with PHA was found to be essential to boost HIV-1 infection of macrophages (Fig. 3D, left panel). Simultaneously, we witnessed efficient virus transmission in the PHA-stimulated CD4+ T cell population, which coincided with p24 release into the cell culture supernatants (Fig. 3D, middle and right panels). Of note, only the lab-adapted NL4-3 was efficiently transmitted to the non-activated CD4+ T cell population.

**Fig 4:**
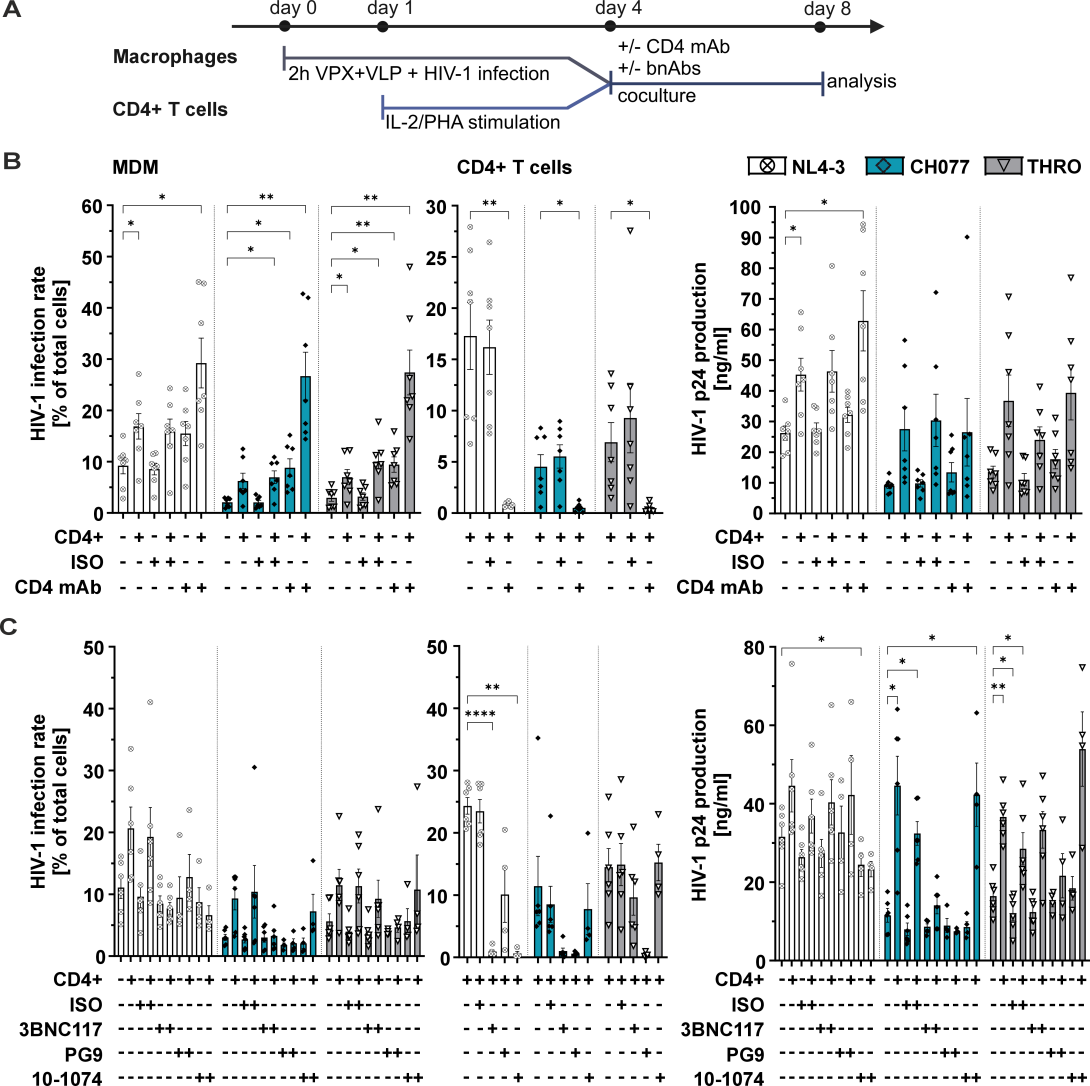
Effect of antibody treatment on T cell-mediated enhancement of macrophage HIV-1 infection. (A) Schematic representation of the experimental design. Setup is similar to Fig. 2A; however, MDMs were additionally treated with either a CD4 antibody or bnAbs during the coculture. (B) The left panel illustrates the HIV-1 infection rate in MDMs cocultured with PHA-prestimulated CD4+ T cells upon the addition of CD4 mAB (clone: RPA-T4; 10 µg/mL) or respective mouse IgG1 isotype control (ISO; 10 µg/mL). The middle panel displays the infection rates in the cocultured CD4+ T cells, and the right panel shows HIV-1 p24 production in the coculture supernatants. Data from *n* = 7 donors are presented as mean ± SEM. Statistical significance was assessed using a paired two-way ANOVA. Dunnett’s multiple comparisons test was applied for MDM and p24 values, while Sidak’s correction was used for CD4+ T cell data. (*P ≤ 0.05 and **P ≤ 0.01). (C) The left panel depicts the HIV-1 infection rate in MDMs cocultured with CD4+ T cells upon bnAb addition (3BNC117, PG9, and 10-1074, 10 µg/mL). A human IgG1 isotype control (ISO; 10 µg/mL) was used as a negative control. The middle panel shows the respective infection rates in the cocultured CD4+ T cells, while the right panel highlights HIV-1 p24 production in the coculture supernatants. Data from *n* = 6 donors for 3BNC117 and *n* = 4 donors for PG9 and 10-1074 are presented as mean ± SEM. Statistical significance was evaluated using a paired mixed-effects analysis with Dunnett’s multiple comparisons test (*P ≤ 0.05, **P ≤ 0.01, and ****P ≤ 0.0001).

We next hypothesized that interactions between the viral envelope glycoprotein GP120 and its receptor CD4 could play a role in enhancing viral replication in macrophages (Fig. 4A). To test for this, we utilized the anti-human CD4 antibody clone RPA-T4, known to bind to CD4, block the interaction with GP120, and suppress T cell activation (55). RPA-T4 was added to 4-day HIV-1-infected macrophages for 2 h before coculture with PHA-activated and non-infected CD4+ T cells. Following the 2-h pre-incubation, the RPA-T4 antibody was maintained continuously throughout the entire coculture period. As controls, we utilized an isotype control or RPA-T4-treated macrophages without the addition of CD4+ T cells. Four days after antibody treatment/coculture, the number of HIV-1 infected macrophages, CD4+ T cells, and HIV-1 p24 production were quantified (Fig. 4B). Surprisingly, treatment with RPA-T4 CD4 antibody only increased HIV-1 production in macrophages and phenocopied the enhancing effect of CD4+ T cell coculture (Fig. 4B, left panel). Furthermore, combining RPA-T4 treatment with non-infected CD4+ T cell coculture had a synergistic effect and resulted in sustained and high levels of HIV-1-infected macrophages, reaching similar infection rates when using the lab-adapted strain NL4-3 compared to the primary isolates CH077 and THRO (Fig. 4B, left panel). As expected, RPA-T4 blocked the transfer of HIV-1 from infected macrophages to non-infected CD4+ T cells. This suggests that the enhancing effect of CD4+ T cell coculture on HIV-1 infection in macrophages is not primarily attributed to virus amplification in the CD4+ T cell fraction. Instead, these findings support a scenario where non-infected activated CD4+ T cells boost macrophage HIV-1 infection through a multilayered cellular mechanism, potentially involving the CD4:TCR to MHCII axis.

Broadly neutralizing antibodies (bnAbs) targeting the viral glycoprotein potently inhibit T cell infection and cell-to-cell spread (56). We sought to determine whether bnAbs also interfere with the CD4+ T cell-mediated enhancement of HIV-1 replication in macrophages. To investigate this, we followed the same experimental setup as previously described. Various bnAbs, including 3BNC117 (targeting the CD4 binding site [57]), PG9 (targeting the V1/V2 region [58]), and 10-1074 (targeting the V3 glycan site [59]), or the respective isotype control was added to 4-day HIV-1-infected macrophages 2 h prior to the addition of autologous non-infected CD4+ T cells (Fig. 4A). After 4 days, quantification of HIV-1 infection rates revealed that bnAbs differentially affected the CD4+ T cell-mediated enhancement of macrophage infection, with effects strongly dependent on the viral strain used (Fig. 4C). All utilized bnAbs reduced CD4+ T cell infection within the coculture of the lab-adapted strain HIV-1 NL4-3, with 3BNC117 and 10-1074 achieving complete neutralization, while PG9 only partially impaired the transmission of HIV-1 to CD4+ T cells. In contrast, the primary isolates CH077 and THRO were resistant to neutralization by 10-1074, and THRO was also partly resistant toward 3BNC117. Importantly, PG9 was the only bnAb that effectively neutralized the transmission of primary HIV-1 strains from macrophages to CD4+ T cell (Fig. 4C, middle panel). While bnAb treatment blocked the cell-to-cell spread of HIV-1 from macrophages to CD4+ T cells, it did not affect HIV-1 replication in macrophage monocultures. Interestingly, when bnAbs neutralized the transmission to CD4+ T cells, the typical enhancement of HIV-1 replication in macrophages, observed under untreated or isotype control conditions, was also absent (Fig. 4C, left panel). This phenotype was consistent with the HIV-1 p24 production measured in the supernatant (Fig. 4C, right panel). These findings emphasize that bnAb treatment not only prevents HIV-1 transmission from macrophages to CD4+ T cells but also disrupts the CD4+ T cell-mediated enhancement of HIV-1 replication in macrophages. Additionally, certain bnAbs may be significantly impaired in their ability to neutralize HIV-1 transmission from infected macrophages to CD4+ T cells.

### CD4+ T cells induce the formation of VCCs in HIV-1-infected MDM, correlating with virus replication

**Fig 5:**
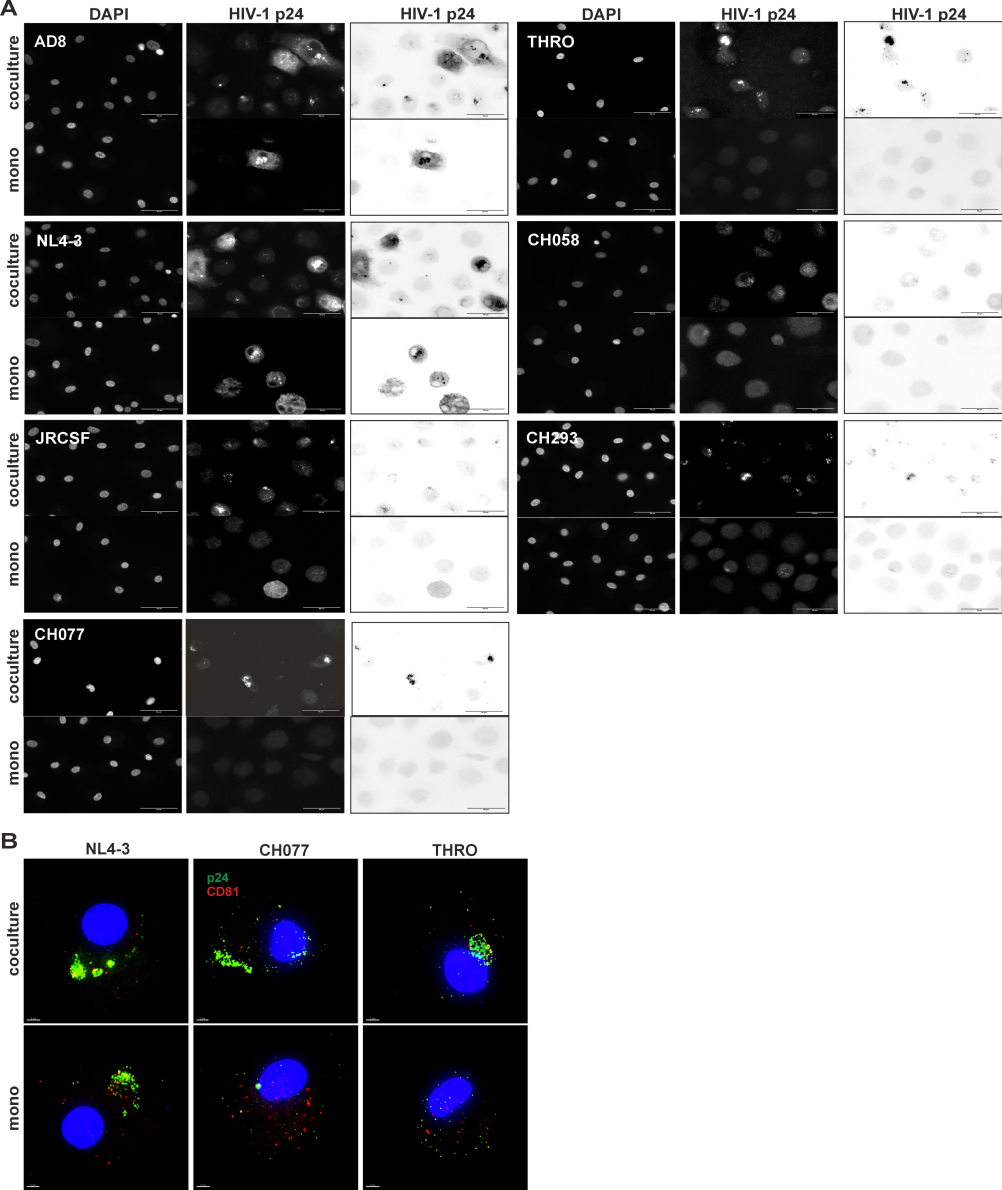
Formation of VCCs in HIV-1-infected MDM with or without CD4+ T cell coculture. (A) MDMs were infected with 25 ng of HIV-1 p24 protein for 16 h, and washed and activated CD4+ T cells were added at 4 dpi in a ratio of 1:10 (1 MDM: 10 CD4+ T cells). At 8 dpi, MDMs were immunostained to detect HIV-1 p24 analyzed by fluorescence microscopy. A 40× magnification objective was used to analyze VCC formation in the cultures. DAPI was used to identify nuclei and individual cells; p24 staining was used to detect infected cells, and VCCs are shown as regular and inverted channels. The scale bar in the images represents 50 µm. The images are representative from one donor out of three donors for CH058, and four donors for AD8, NL4-3, JRCSF, CH077, THRO, and CH293. (B) MDMs were infected with 250 ng of HIV-1 p24 on cover slips for 16 h, and activated CD4+ T cells were added at 4 dpi in a ratio of 1:10 (1 MDM: 10 CD4+ T cells). At 8 dpi, MDMs were immunostained to detect HIV-1 p24 and analyzed by fluorescence microscopy. MDMs were harvested, permeabilized, and immunostained for HIV-1 p24 and CD81. DAPI was used to counterstain the nuclei. Microscopy was performed using a 60× magnification objective. The red channel represents CD81, and the green channel represents p24. The scale bar represents 5 µm. Representative images for NL4-3, CH077, and THRO from one out of two donors are shown.

**Fig 6:**
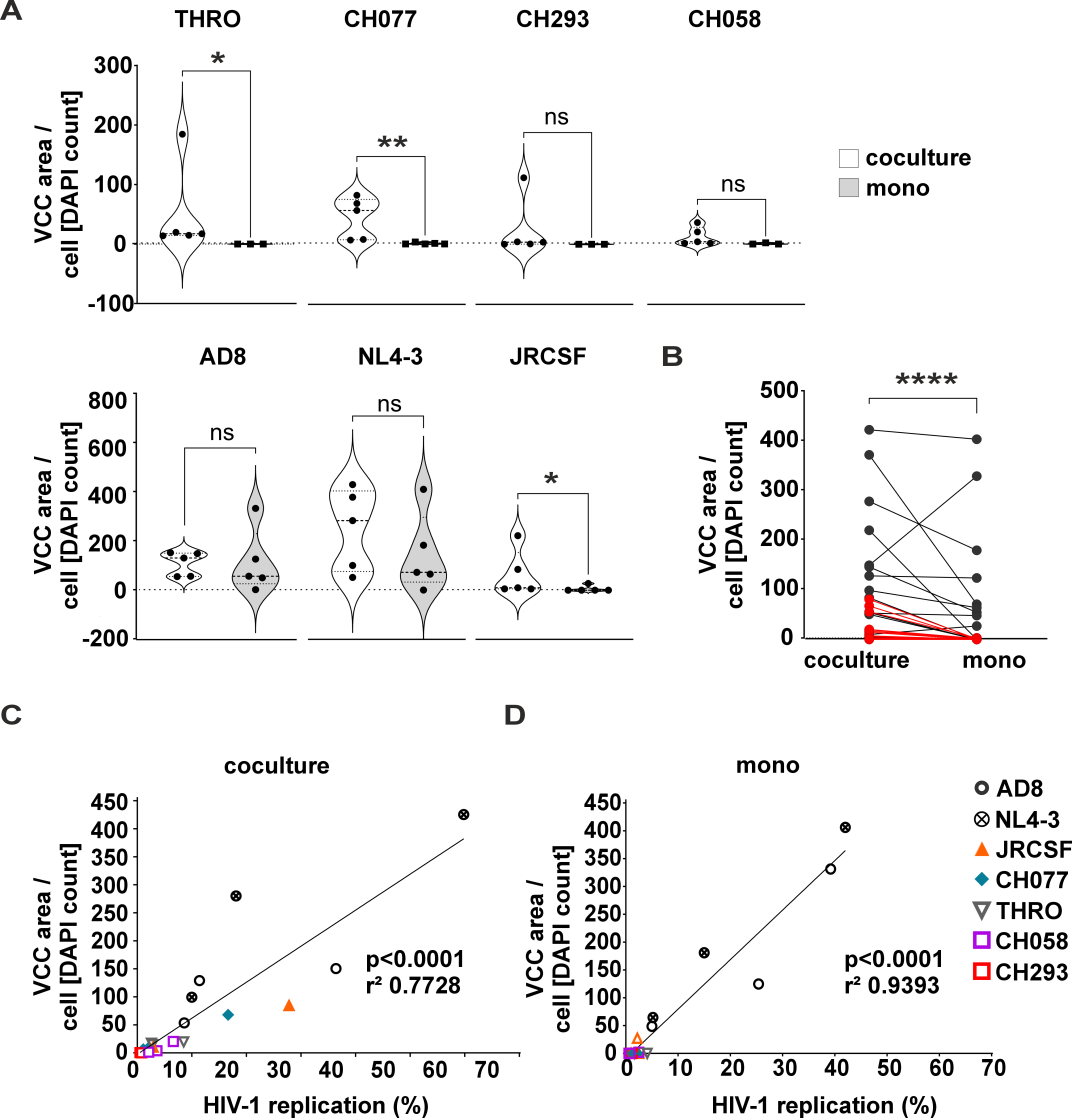
Non-infected CD4+ T cells induce the formation of VCCs in HIV-1-infected MDM, which correlates with virus replication. Monocyte-derived macrophages were infected with 25 ng of HIV-1 p24 protein for 16 h, washed, and subsequently activated CD4+ T cells were added at 3 dpi in a ratio of 1:10 (1 MDM: 10 CD4+ T cells). At 7 dpi, MDMs were immunostained to detect HIV-1 p24 followed by counterstaining with DAPI and analysis via fluorescence microscopy at 40× magnification. (A) Calculation of VCCs area sum over DAPI counting in MDM from coculture system (white violins) and MDM cultured alone (gray violins) considering each HIV-1 strain. Five donors for coculture system for all virus strains, and for monoculture system, three donors of CH058, THRO, and CH293 and five donors of AD8, JRCSF, NL4-3, and CH077. Significance was tested with unpaired *t*-test and Mann-Whitney test. *P < 0.05; **P < 0.01; and ns, not significant. (B) The ratio of VCC area sum over DAPI counting is expressed comparing each system (coculture and monoculture) independently of the HIV-1 strain (black spots represent laboratory-adapted strains, and red spots represent the primary strains, *n* = 21, i.e., three biological replicates of each of the seven HIV-1 strains). Significance was tested with paired *t*-test and Wilcoxon matched-pairs signed rank test. ****P < 0.0001. (C and D) Correlation between the VCC area sum over DAPI counting and infection rate (expressed as HIV-1 replication rate in percentage) in (C) MDM from coculture and (D) MDM cultured alone. *n* = 3 donors. The trendline shows a linear regression analysis with squared Pearson correlation coefficient (*r*^2^) and calculated statistical significance.

HIV-1 infection of macrophages leads to the sequestration of newly formed viruses in VCCs, variously characterized as a source of virus for trans-infection, a site of virus assembly, or a site of virus storage following assembly on the plasma membrane (12, 20, 60, 61). However, up to now, VCC formation has been exclusively studied in lab-adapted strains NL4-3 and AD8 (12, 14, 15, 20, 23, 60–63). Hence, we first evaluated the ability of primary HIV-1 strains to form VCCs in macrophages. As expected and in line with the inability of these strains to initiate productive infection in MDMs (Fig. 1B), we observed few to no VCCs when stained for intracellular p24 (Fig. 5A). We then hypothesized that coculture of infected macrophages with autologous non-infected CD4+ T cells might, apart from enhancing macrophage infection rates, facilitate formation of VCCs. To address this, HIV-1-infected MDMs cocultured with non-infected CD4+T cells were stained against p24 in order to visualize the amount of VCCs and their subcellular distribution. Compared to macrophages infected and cultured alone, we observed the appearance of VCCs in macrophages infected with primary HIV-1 strains upon coculture with CD4+ T cells. However, these VCCs appeared fewer and less prominent compared to those formed by lab-adapted strains (Fig. 5A), a phenotype we confirmed by high-resolution microscopy (Fig. 5B). Quantifying the total VCC area normalized to cell numbers (by counting the DAPI+ nuclei), the previous qualitative assessment became strikingly clear (Fig. 6A). Indeed, neither JRCSF nor one of the other T/F and chronic strains were able to establish VCCs in MDM, whereas coculture with autologous non-infected CD4+ T cells initiated VCC formation. In contrast, the amount of VCCs was nearly identical when MDMs were initially infected with AD8 or NL4-3 irrespective of adding autologous non-infected CD4+ T cells to the cultures or not (Fig. 6A). Overall, coculturing HIV-1-infected MDM with autologous non-infected CD4+ T cells robustly induced the formation of VCCs (Fig. 6B).

As previously mentioned, VCCs may serve as storage compartments of HIV-1 in macrophages or play more direct roles in HIV-1 trans-infection or spread. Given that coculture induced replication of primary strains in MDM and promoted VCC formation, we hypothesized that these two phenotypes might be interconnected. Interestingly, the VCC area correlated with HIV-1 replication rate in all the T/F strains (THRO, CH058, and CH077), the HIV-1 chronic strain (CH293), the primary strain JRCSF, as well as the lab-adapted ones (AD8 and NL4-3) either in macrophages from cocultures or macrophages cultured alone (see Fig. 6C and D, respectively).

Altogether, these results demonstrate that replication of primary HIV-1 strains in macrophages is strongly increased by coculture with non-infected CD4+ T cells, indicating that these strains depend on cellular cross-talk for efficient transmission and spread. Furthermore, coculture with autologous non-infected CD4+ T cell induces VCC formation in macrophages, and the abundance of VCCs correlates with increased virus replication.

## DISCUSSION

A major challenge in HIV-1 therapy and cure approaches is the management of long-term viral persistence in cells that act as a reservoir of the virus, including CD4+ memory T cells and tissue-resident macrophages. Hence, there is a strong interest in understanding modes of viral transmission and reactivation of virus replication in those cells (64–66). On top of that, it is essential to include primary HIV-1 isolates, such as T/F or chronic HIV-1 strains, alongside different lab-adapted strains in such studies.

Previous studies have assessed how these isolates replicate in CD4+ T cells and macrophages and found, in accordance with our data, that T/F viruses are inefficient in infecting macrophages, even though they are capable of using CCR5 as a co-receptor. However, their replication is readily detectable in CD4+ T cells (30, 33, 47). This block in macrophage infection can be overcome by cell-to-cell spread, where HIV-1 is directly transferred from CD4+ T cells to macrophages (18, 22, 24, 25, 47, 67, 68). In addition, through processes like transcytosis, antigen-presenting cells, including macrophages and DCs, capture infectious viruses to pass them on for CD4+ T cell infection (69). Also, the cellular environment generated after macrophage infection with lab-adapted or T/F HIV strains can enhance the infection of resting CD4+ T cells (28) and can even skew the differentiation of activated CD4+ T cells into more permissive profiles (29). Vice versa, it is well established that HIV-1-infected CD4+ T cells transmit HIV-1 to macrophages through the virological synapse, direct cell-to-cell contact, and even via phagocytosis and fusion with infected CD4+ T cells (24, 70–72).

In contrast, we are not aware of studies that investigate if the fate, respectively, course of HIV-1 replication in macrophages is influenced by coculture with autologous non-infected CD4+ T cells (24). Our findings highlight that lab-adapted strains replicate efficiently in macrophages independent of CD4+ T cell coculture, whereas replication of primary HIV-1 strains in macrophages is recovered and strongly fueled by the addition of non-infected CD4+ T cells. Direct cellular interaction is required for this phenotype, and the non-infected CD4+ T cells need to be pre-activated (Fig. 3C and D). Strikingly, our data also argue against a scenario in which non-infected CD4+ T cells enhance macrophage infection by viral replication and re-transmission. This is evident by the addition of CD4 mAb RPA-T4, which completely blocks infection of cocultured non-infected CD4+ T cells while further enhancing infection (Fig. 4B). RPA-T4 is known to block the CD4 interaction site with GP120 and suppress T cell activation (55). Therefore, while it is difficult to reconcile how this CD4 mAb phenocopies the effect of non-infected CD4+ T cell coculture, it seems that the CD4:MHCII axis, as well as other T cell-to-macrophage interactions, might be involved. Future studies will be necessary to delineate the exact mechanisms underlying this phenomenon. One hypothesis we already addressed is a potential reduction of the HIV-1 restriction factor SAMHD1 in macrophages (42), upon coculture with non-infected CD4+ T cells or treatment with the CD4-mAb RPA-T4, which was not the case (Fig. S1).

Another key aspect of this study is the differential inhibitory activity of neutralizing antibodies. Independent of their neutralizing activities, bnAb treatment did not affect the infection rates in MDM monocultures. Given that HIV-1 accumulates in intracellular VCCs within MDM, this finding aligns with previous studies showing that VCCs are largely inaccessible to bnAbs, effectively shielding the virus from the host’s immune response (15, 19, 73). Furthermore, among the three bnAbs tested, only PG9, which targets the V1/V2 glycan shield of GP120 (58), effectively inhibited virus transmission from macrophages to non-infected CD4+ T cells while concomitantly suppressing T cell-mediated enhancement of macrophage infection (Fig. 4C). This was only evident for the two primary strains tested, with PG9 showing limited neutralization capacity against the lab-adapted strain NL4-3. On the contrary, 3BNC117 and 10-1074, which target the GP120 CD4 binding site and the V3 glycan (57, 59), respectively, showed complete neutralization of CD4+ T cell infection within cocultures for the lab-adapted strain NL4-3 and also inhibited the T cell-mediated enhancement of macrophage infection. However, these two bnABs failed to neutralize the transmission of at least one of the primary isolates, CH077 and THRO, highlighting the strain-specific variability in bnAb efficacy when it comes to cell-to-cell transmission (74, 75). This underscores the importance of considering the viral strain and transmission mode when evaluating bnAbs for therapeutic use. Strategies targeting bnAbs must account for their variable efficacy in different cellular environments, particularly those involving macrophage reservoirs and cell-to-cell transmission. Furthermore, bnAbs that efficiently suppress cell-free HIV-1 infection of CD4+ T cells or interfere with viral cell-to-cell transmission (56) might be compromised in their ability to suppress CD4+ T cell-mediated enhancement of HIV-1 macrophage infection.

Regardless, it became clear in our experiments that viral replication in macrophages is intimately linked to the formation of macrophage internal virus-containing compartments. VCCs are vacuolar subcellular structures that can be connected to the plasma membrane where it has been described that HIV-1 accumulates as infectious virions (12, 14, 15, 23, 63, 76). Macrophage internal HIV-1 in VCCs is shielded from recognition by broadly neutralizing antibodies (15, 73), and VCCs are characterized as storage and virus transmission compartments (8, 18, 23, 62). Here, we extended previous results showing the presence of VCCs in macrophages infected with not only lab-adapted HIV-1 strains but also with T/F and chronic strains, which had not been studied before. While lab-adapted HIV-1 strains readily form VCCs, primary strains require the presence of non-infected CD4+ T cells to replicate in macrophages and form VCCs, suggesting a crucial role for T cells in establishing macrophage reservoirs. The fact that VCCs in macrophages seem to represent an immune-privileged niche refractory to neutralizing antibodies (15, 73) underscores the challenge posed by the complex membrane architecture. These structures create a viral hideout that poses an obstacle for HIV-1 elimination strategies and highlights the necessity to purge this reservoir through therapeutic intervention (19, 64). Of note, evidence suggests that virus release from macrophages is impaired when VCC-anchored actin filaments are disrupted via experimental drugs (77).

In summary, our results suggest that primary patient-derived HIV-1 strains form VCCs in macrophages upon coculture with autologous non-infected T cells and that this process correlates with virus replication. This underscores an essential role for VCCs in the replication of patient-derived HIV-1 in macrophages, which is fueled by non-infected CD4+ T cells in a CD4- and GP120-dependent manner. Considering the important role of macrophages as reservoir cells, it will be essential to develop strategies that target HIV-1 in VCCs and VCC formation or destroy VCCs in conjunction with therapeutic interventions that aim to reduce the burden of latent HIV-1 in CD4 +T cells. Only by comprehensively addressing HIV-1 persistence in the variety of potential reservoirs, the current ambitious goal of an HIV-1 functional cure might become achievable.

## Data Availability

All data generated and analyzed during this study are included in this published article.

## SUPPLEMENTAL MATERIAL

Figure S1 jvi.00182-25-s0001.pdf CD4+ T cell coculture or CD4 antibody treatment does not modulate SAMHD1 in primary macrophages.

ASM does not own the copyrights to Supplemental Material that may be linked to, or accessed through, an article. The authors have granted ASM a non-exclusive, world-wide license to publish the Supplemental Material files. Please contact the corresponding author directly for reuse.
