# Supplementary material for: CD4+ T cells facilitate replication of primary HIV-1 strains in macrophages and formation of macrophage internal virus-containing compartments
Source: J Virol. 2025 Mar 25;99(4):e00182-25. doi: 10.1128/jvi.00182-25 (PMC11998544; doi:10.1128/jvi.00182-25)
Supplement: Figure S1 — CD4+ T cell coculture or CD4 antibody treatment does not modulate SAMHD1 in primary macrophages. [file jvi.00182-25-s0001.pdf]

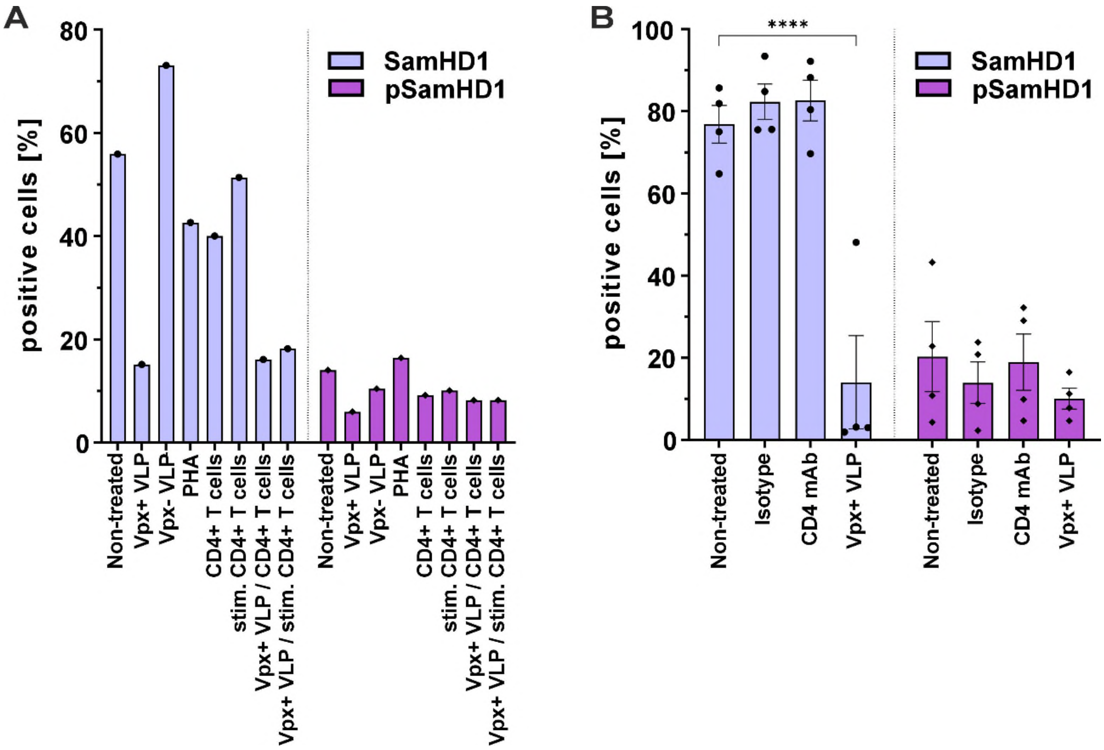

Figure S1. CD4+ T cell coculture or CD4 antibody treatment does not modulate SAMHD1 in primary macrophages. (A) MDMs were cocultured with CD4+ T cells for 4 days, followed by harvesting of MDMs to assess SamHD1 and phosphorylated SamHD1 levels by flow cytometry. As a control, VPX-containing VLPs were used, which were either added 4 days prior to the addition of CD4+ T cells or along with the T cells. MDMs were treated with VPX on day 1, and CD4+ T cells were cocultured with infected MDMs at a 1:10 ratio (1 MDM: 10 CD4+ T cells) on day 4. SamHD1 and phosphorylated SamHD1 data are plotted as percentage of positive cells, with data from n=1 donor presented. (B) MDMs were treated with CD4-mAb (clone: RPA-T4; 10 µg/ml) or the respective mouse IgG1 isotype control (ISO; 10 µg/ml). VPX-containing VLPs were used as a positive control for SamHD1 modulation. Data are plotted as the percentage of positive cells, with data from n=4 donors presented as mean ± SEM. Statistical significance was tested using a paired two-way ANOVA with Dunnett's multiple comparisons test (**** $p \leq 0.0001$).
